# Seroprevalence against measles, Austria, stratified by birth years 1922 to 2024

**DOI:** 10.2807/1560-7917.ES.2025.30.16.2400684

**Published:** 2025-04-24

**Authors:** David N Springer, Christian Borsodi, Jeremy V Camp, Monika Redlberger-Fritz, Heidemarie Holzmann, Michael Kundi, Judith H Aberle, Karin Stiasny, Lukas Weseslindtner

**Affiliations:** 1 Center for Virology, Medical University of Vienna, Vienna, Austria; 2 Center for Public Health, Medical University of Vienna, Vienna, Austria

**Keywords:** measles, seroprevalence, Austria, antibody, waning, measles–mumps–rubella (MMR) vaccine, vaccines and immunisation

## Abstract

**Background:**

Vaccination programmes initiated in the early 1970s reduced the incidence of measles in Austria, which resulted in the interruption of endemic measles virus (MeV) circulation and the achievement of elimination status in 2018. However, large outbreaks occurred in 2023 and 2024.

**Aim:**

By assessing MeV-specific IgG antibody levels, we analysed if immunity recently declined due to the COVID-19 pandemic, vaccine-induced immunity waned over long term or immunity gaps already pre-existed in the population.

**Methods:**

We determined anti-MeV antibody levels in a retrospective dataset of 56,360 diagnostic samples (from 50,754 individuals) collected 2010–2024 and correlated antibody cutoffs to titres from a live-virus neutralisation test.

**Results:**

Individuals born before 1970 (n = 15,007) had antibody levels > 3,000 IU/L, persisting into higher age, and < 2% (n = 300) of them were seronegative. In contrast, individuals born after 1990 (n = 12,778) displayed seronegativity rates of 13–20% and lower median antibody concentrations in seropositive individuals (449–773 IU/L). In these individuals, antibody levels decreased noticeably between the ages of 2 and 10 years but remained stable between those aged 10 and 30 years. There was no significant difference in seronegativity rates at the age of 12–24 months in children born 2016–2019 and 2020–2022 (the years of the COVID-19 pandemic).

**Conclusion:**

In Austria, there are significant immunity gaps in individuals born after 1970, which pre-existed before the COVID-19 pandemic. Thus, young and middle-aged populations not immune against measles should be vaccinated to counteract a further decline of immunity at the population level and prevent outbreaks whenever MeV is imported.

**Key public health message**

**What did you want to address in this study and why?**

In 2023 and 2024, measles outbreaks with 728 infections occurred in Austria. Therefore, we investigated immunity against measles virus in the Austrian population. We analysed antibodies against measles virus in blood samples collected 2010–2024 from 50,754 individuals, then compared the antibody levels among different age groups and finally analysed whether the COVID-19 pandemic had an impact on immunity against measles.

**What have we learnt from this study?**

Almost all individuals born before 1970 were immune against measles, likely due to an infection in childhood when the virus circulated frequently and caused disease, including severe forms. Many individuals born after 1990, when there was less virus circulation and immunity was mainly acquired by vaccinations, had no or only insufficient immunity against measles virus. The pandemic had no impact on measles immunity in Austrian children.

**What are the implications of your findings for public health?**

Measles vaccination protects against the disease and severe forms. Accumulating numbers over years of young and middle-aged individuals not having protective immunity against measles virus might have contributed to why measles outbreaks recently occurred in Austria in addition to affecting mainly unvaccinated individuals. There is an urgent need to increase the vaccination rates in individuals who are not sufficiently immune to measles.

## Introduction

While measles was widespread in the pre-vaccination era, several European countries, including Austria, have achieved elimination status in recent decades with only sporadic outbreaks occurring [1-4]. In 2023, however, 186 laboratory-confirmed measles cases were notified in Austria, corresponding to an incidence of 20.7 per 1,000,000 inhabitants, the third highest in Europe in 2023 [5,6]. Then, in the first half of 2024 alone, 445 laboratory-confirmed measles cases were notified, caused by multiple introductions of at least 17 different measles virus (MeV) strains [7]. These cases occurred in all nine federal provinces of Austria and in persons of varying ages (0–68 years), affecting a relatively broad population range. Most of these were primary infections in unvaccinated individuals, whereas 18 (7%) of 268 infected patients from 2023 and 2024 (with available sera) displayed MeV-specific antibodies of high avidity, indicating possible reinfection after prior vaccination(s). More detailed information on the age distributions and the results of avidity testing are presented in Supplementary Figure S1.

Thus, questions have been raised whether these outbreaks occurred because of a recent decrease in vaccination coverage during the COVID-19 pandemic [8], a waning of protective antibody levels in vaccinated individuals over time [9] or pre-existing immunity gaps in specific population groups [10]. Additionally, the optimal time point to recommend the first vaccination in children has been reconsidered, balancing the need to reduce the number of measles-susceptible infants against the risk of compromising vaccine efficacy if measles vaccinations are administered too early, as high levels of maternal antibodies transferred to the fetus during pregnancy might impair the infants’ vaccine responses [11]. Therefore, recommendations should consider the expected levels and kinetics of maternal antibodies in infants during their first year of life which depend on the anti-MeV antibody levels in pregnant people.

To address these questions, we assessed seroprevalence of MeV antibodies in Austria and matched IgG antibody levels against MeV and rubella virus (RuV) to estimate the proportion of the study cohort fully vaccinated with two doses of trivalent MMR (measles–mumps–rubella) vaccines.

## Methods

### Measles–mumps–rubella immunisation programmes in Austria

Nationwide immunisation programmes against measles and mumps were initiated in Austria in 1974, using a single dose of a vaccine containing live-attenuated measles virus (MCV). Between 1974 and 1993, a bivalent MCV (measles–mumps vaccine) was offered to all children at 15 months of age, while vaccination against rubella was only recommended for females between 1984 and 1993. Since 1994, two doses of the MMR vaccine have been recommended for all individuals, regardless of sex. More details of the measles vaccination history are presented in Supplementary Table S1. Since 2018, the first dose of MMR vaccine is recommended at 9 months of age and the second dose at least 3 months later (or 4 weeks later in individuals aged > 12 months at the first dose).

### Serum samples

Serum samples were sent to the diagnostic laboratory of the National Reference Laboratory (NRL) for measles by hospitals, laboratories and physicians from different parts of Austria. Commercial, CE-certified MeV IgG (and IgM) ELISA tests were performed as part of routine diagnostic serology (see more details in Quantification of measles virus-specific antibodies). Of note, the same anti-MeV-IgG antibody test kit was applied from 1 January 2010 until the study was initiated on 1 April 2024. Therefore, the study could include all available anti-MeV-IgG-antibody test results from this period. Supplementary Figure S2 shows the detailed inclusion and analysis plan. Patient data and test results were stored in the electronic database of the NRL.

### Quantification of measles virus-specific antibodies

Measles virus-specific serum IgG and IgM antibody concentrations were quantified using a commercial ELISA (Anti-Measles Virus ELISA IgG and IgM, Euroimmun, Lübeck, Germany) following the manufacturer's instructions.

For the qualitative interpretation of the ELISA results, we used the cutoff levels recommended by the manufacturer: negative: < 150 IU/L, borderline: 150–199 IU/L and positive: ≥ 200 IU/L. Antibody levels above the quantification range (≥ 5,000 IU/L) were set to 5,000 IU/L. Positive samples with IgG levels ≥ 200 IU/L were further (arbitrarily) stratified as 200–275 IU/L, 276–1,400 IU/L, 1,401–3,500 and > 3,500 IU/L for a better visualisation of the results.

To evaluate the correlation between quantitative ELISA results and the neutralising activity of the MeV-specific antibodies, we performed a MeV live-virus neutralisation test (NT) using a protocol described previously [12] and in the Supplementary Material.

### Data pseudonymisation, cleaning of patient records and definition of cohorts

Patient information was extracted from the database of the NRL and pseudonymised for further analysis, following a protocol approved by the local ethics committee (EK1452/2024).

The initial data export included all test results for all samples tested for diagnostic purposes between 1 January 2010 and 1 April 2024 (n = 61,792). For all further analyses, the blood sampling date was used. When the exact sampling date was unavailable, we used the date of sample registration. Results from patients were excluded when an acute MeV infection was likely, i.e. individuals with a positive or borderline IgM result, a positive PCR result and/or samples referred with an explicitly declared clinical suspicion for measles (n = 1,360). Details on the data cleaning process are presented in Supplementary Figure S2.

Next, test results from patients with immunosuppression were excluded based on clinical data (e.g. after organ transplantation, under chemotherapy or with autoimmune disorders; n = 3,639). Six individuals born before 1922 were excluded because of the low number of individuals in this age group. In addition, samples with missing data (e.g. date of birth) or invalid test results were excluded (n = 427). Follow-up samples (n = 5,606) from 4,020 individuals were excluded from the primary cohort but were used in a separate longitudinal analysis. This resulted in a final study cohort of 56,360 samples from 50,754 individuals, all tested using the same anti-MeV-IgG antibody assay, and the age range was 0–94 years at the time of the antibody test.

For the cross-sectional analyses, only the first available test result was considered for each individual tested. Children aged < 2 years (n = 725, with 201 aged < 9 months) were also excluded and analysed separately. Additionally, we analysed a subset of the study cohort of 1,692 pregnant people (unrelated to the infant cohort). Follow-up samples (n = 5,606) from 4,020 individuals were excluded from the main cohort but were used in a separate longitudinal analysis. This longitudinal cohort was further stratified to only include the first and last available sample of initially seropositive individuals born before 1965 (immunity likely due to measles infection, n = 296) or born after 1990 (immunity likely due to vaccination, n = 872). The inclusion and exclusion processes are outlined in Supplementary Figure S2.

### Statistical analysis

Statistical analyses were performed using R statistical programme version 4.2 (https://www.r-project.org/). First, we analysed the correlation between anti-MeV-IgG antibody levels and log2-transformed neutralisation (NT) titres in a subset of samples in which both tests were conducted, using linear regression and the Pearson correlation coefficient. Additionally, we performed a receiver operating characteristic (ROC) analysis to determine the sensitivity and specificity of the recommended cutoffs for the commercial anti-MeV-IgG ELISA to detect positive NT results (titre ≥ 10). The titre ≥ 10 corresponded to ≥ 120 IU/L after calibration to the World Health Organization (WHO) the National Institute for Biological Standards and Control (NIBSC) 97/648 3rd International Standard, previously described as a correlate of protection against symptomatic measles [13].

For the age-stratified seroprevalence analysis, results of the anti-MeV-IgG testing were grouped according to the year of birth of the tested individuals in 4-year brackets, denoted as, e.g. 1930–1934 (i.e. regardless of the sampling date). Then, the number and percentage of IgG-positive, borderline and negative results of tests of these individuals for these different birth year strata were calculated, and the median and interquartile ranges of the quantitative IgG levels for the various age groups (in IU/L) were recorded. Furthermore, antibody concentrations and seronegativity rates were compared among birth decades (< 1970, 1970–1979, 1980–1989, 1990–1999, 2000–2019, ≥ 2020) using the Kruskal-Wallis test followed by multiplicity adjusted Dunn’s test and pairwise chi-square tests, respectively. Likewise, similar analyses of qualitative and quantitative anti-MeV-IgG results were conducted separately for males and females, pregnant people and children aged < 2 years and 9 months. In the latter, a locally estimated scatterplot smoothing (LOESS) regression was used to visualise the waning of maternal antibody levels (cross-sectional analysis, i.e. one sample per individual).

Next, we investigated a possible waning of anti-MeV-IgG levels after immunisation in cross-sectional and longitudinal analyses (follow-up sera). Here, individuals born before 1965 and after 1990 were analysed separately. Median antibody levels and the seropositivity rates were calculated and stratified by age, and trends were visualised using LOESS regression. For the longitudinal analysis, the first sample for each individual was set as the baseline time point, and individual antibody kinetics were visualised over time. Additionally, the difference between the antibody levels of the last available sample and the first positive sample was calculated and analysed by linear regression to estimate the rates of antibody waning, thereby only including one data point per individual.

The proportion of unvaccinated individuals among the measles seronegative population was estimated by analysing the subset of participants born ≥ 1994 (when MMR vaccinations were first implemented in Austria) and for whom paired measles and rubella IgG results were available. Seronegativity for both viruses (stratified as percentages by birth year) served as a proxy for non-vaccinated individuals. The detailed methodology is presented in Supplementary Material.

## Results

### Correlation of quantitative ELISA antibody measurements with measles virus neutralising antibody titres

First, we re-evaluated the correlation of the quantitative ELISA IgG antibody results with the NT results, using a subset of 481 samples for which paired ELISA and MeV-NT results were available. There was a significant correlation of the anti-MeV-IgG antibody units (in IU/L) with MeV-NT titres (Pearson r = 0.62; p < 0.001). The details can be seen in Supplementary Figure S3. For the detection of MeV-specific neutralising activity (i.e. MeV-NT titre ≥ 10), the cutoff between a positive and borderline interpretation of > 200 IU/L corresponded to the upper-left point determined by Youden's index in a ROC curve (area under the curve (AUC) = 0.95, upper left: 203 IU/L). The cutoffs, sensitivity and specificity can be seen in Supplementary Table S2. Moreover, ELISA results < 150 IU/L, the cutoff of negative results, were strongly predicative of a negative MeV-NT titre (98% sensitivity and 66% specificity for ≥ 120 IU/L calibrated on the WHO NIBSC 97/648 standard).

### Demographic overview

We performed a comprehensive seroprevalence analysis of anti-MeV-IgG levels in different birth year strata in Austria, including 50,754 individuals in all analyses (males: n = 16,311; females: n = 34,254; missing data on sex: n = 189). More details can be seen in Supplementary Tables S3–S4. After stratifying by the birth year (in 4-year intervals), all birth year strata 1942–2017 included > 500 individuals, while the smallest number of individuals per age groups were those born 1922–1925 (n = 40) and 1926–1929 (n = 68).

### Measles seroprevalence in different birth year strata

**Figure 1:**
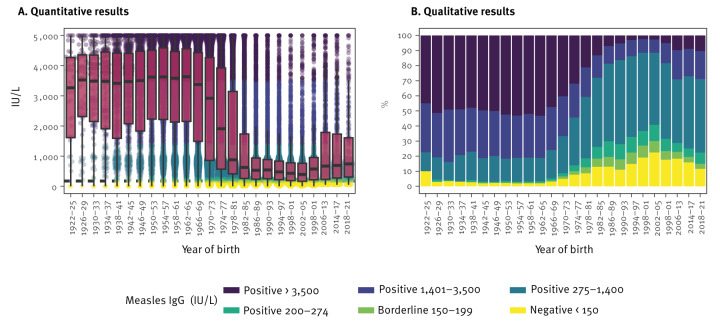
Quantitative and qualitative results of IgG antibodies against measles virus, by birth year, Austria, 2010–2024 (n = 50,029)

First, we analysed anti-MeV-IgG antibody levels in 50,029 individuals aged ≥ 2 years stratified by the birth year (in 4-year intervals) and the decade of birth. High anti-MeV-IgG antibody levels were measured in individuals born before 1970 (n = 15,007; median: 3,535 IU/L; Figure 1A). Results for the decade of birth are presented in Supplementary Figure S4A. In subsequent birth decades until 2009, median antibody concentrations were significantly lower: 2,225 IU/L for 1970–1979 (n = 10,772), 620 IU/L for 1980–1989 (n = 11,472), 502 IU/L for 1990–1999 (n = 7,528) and 449 IU/L for 2000–2009 (n = 3,520) (p < 0.05 for comparisons among consecutive decades respectively). However, median antibody concentrations were significantly higher in those born 2010–2019 (684 IU/L; n = 1,660) than in those born 2000–2009 (449 IU/L; n = 3,520; p < 0.05) and slightly lower compared with those born ≥ 2020, although this did not reach statistical significance (773 IU/L; n = 70; p > 0.05, Kruskal-Wallis and Dunn's multiple comparison test).

The figure displays results from individuals aged 2–93 years (one test per individual).

Panel A. The boxes indicate the median and the quartiles, and the whiskers indicate the range (excluding outliers). The dots represent single sera, and their placement approximates the data distribution.

Panel B. Percentages of positive and negative sample strata per birth year as indicated by the colours provided in the legend.

Accordingly, only 348 (2%) of 15,007 individuals born before 1970 were seronegative for measles, as presented in Supplementary Figure S4B. Seronegativity was 7% (715/10,772) in individuals born 1970–1979, 12% (1,389/11,472) in those born 1980–1989, 13% (1,016/7,528) in those born 1990–1999 and 20% (704/3,520) in those born 2000–2009. Of 1,660 individuals born 2010–2019, 274 (17%) were seronegative and 16% (11 of 70) of those born ≥ 2020 (p < 0.05 for pairwise comparisons of subsequent decades until 2019, and p > 0.05 for 2010–2019 vs. ≥ 2020, chi-square tests, followed by Bonferroni correction).

When we analysed the birth strata (in 4-year intervals), proportions of seronegative individuals increased in samples from those born 1970–2005, except for individuals born 1990–1993 (n = 3,616), where seronegativity was lower (11%) compared with 13% in those born 1986–1989 (n = 4,112). The highest proportions of seronegativity (15% and 22%) were observed in individuals born 1998–2001 (n = 1,897) and 2002–2005 (n = 1,448). After this peak, seronegativity rates declined to 12% in those born 2018–2021 (n = 242) (Figure 1B).

When we compared seronegativity in males with females born after the introduction of the measles vaccination in Austria (i.e. ≥ 1970), we found that seronegativity was higher in males (n = 10,757; 14%) than in females (n = 24,112; 10%) (p < 0.001, chi-square test). The detailed results are presented in Supplementary Figure S5.

### Seroprevalence in pregnant people

**Figure 2:**
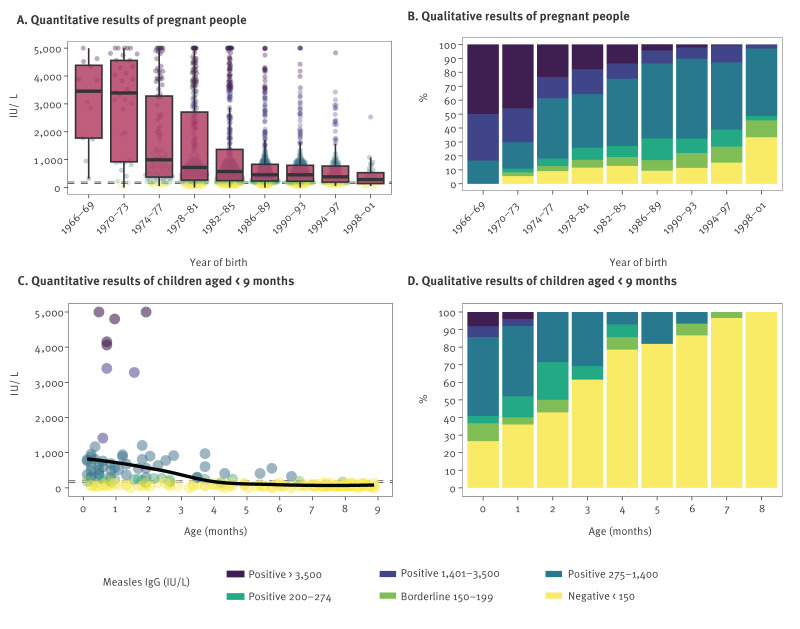
Quantitative and qualitative results of IgG antibodies against measles virus in pregnant people (n = 1,692) and children aged < 9 months (n = 201), Austria, 2010–2024

Maternal antibodies may provide passive immunity to the neonate and influence the measles vaccine efficacy in infants. Therefore, we analysed anti-MeV-IgG antibody levels in pregnant people (n = 1,692). As in the general population, the highest median IgG levels were observed in those born 1966–1969 (n = 12; 3,455 IU/L) and 1970–1973 (n = 37; 3,394 IU/L) (Figure 2A), although this was based on limited sample numbers. Antibody levels were lower in samples from those born 1974–2001 (n = 1,643), varying from 993 IU/L in birth years 1974–1977 (n = 111) to 284 IU/L in birth years 1998–2001 (n = 33) (Figure 2A). Correspondingly, none of the 12 pregnant people born before 1970 were seronegative, but 14–27% of those born 1974–1997 were seronegative, peaking at 33% in those born 1998–2001 (Figure 2B). More details are presented in Supplementary Table S5.

Panel A. The boxes indicate the median, and the quartiles, and the whiskers indicate the range (excluding outliers). The dots represent single sera, and their placement approximates the data distribution.

Panel B. Qualitative results per birth decade.

Panel C. Each dot represents a single serum. The black line displays a non-parametric LOESS regression to visualise the trend.

Panel D. Qualitative results per age in complete months (rounded down).

### Levels of maternal antibodies in infants

To investigate waning of maternal immunity in infants, we analysed anti-MeV-IgG antibody levels in children aged < 9 months (n = 201, one sample per individual), i.e. before the age of the first recommended vaccination in Austria [14]. In the first month after birth, median antibody levels were at 409 IU/L, but at 3 months of age, median antibody levels had declined to 104 IU/L (Figure 2C). Correspondingly, while 22 (30%) of 74 tested infants were seronegative (< 150 IU/L) in the first 2 months after birth, this reached 87% by 6 months of age (13 of 15 infants between 5 and 6 months) (Figure 2D).

### Impact of the COVID-19 pandemic on immunisation rates of children aged 12–24 months

To investigate whether there was a decrease in the immunisation rates due to the COVID-19 pandemic, we compared seroprevalence rates in children aged 12–24 months stratified by their birth year. Details are presented in Supplementary Figure S6. We found that children born shortly before or during the COVID-19 pandemic (July 2019–December 2022, n = 102) had a similar seronegativity rate (23%) compared with those born in the years before the pandemic (2016–June 2019: n = 93; 22% seronegativity; p = 0.86, chi-square test).

### Waning of measles virus-specific IgG antibodies over time

**Figure 3:**
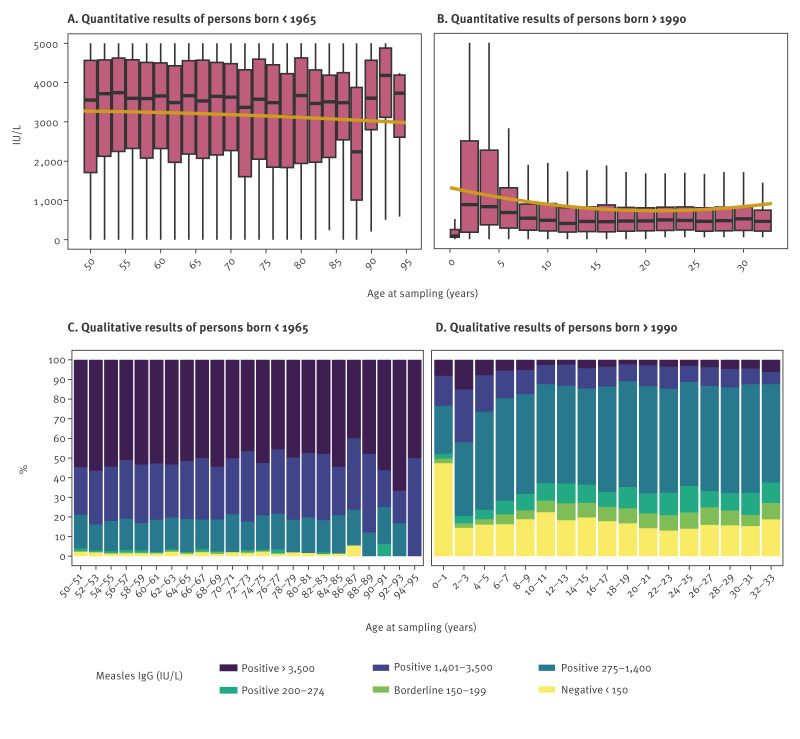
Quantitative and qualitative results of IgG antibodies against measles virus in individuals born < 1965 (n = 10,533) and > 1990 (n = 12,502), Austria, 2010–2024

We tested whether anti-MeV-IgG antibody levels wane in previously immune individuals over time by stratifying antibody test results by patient age at blood sampling in the sub-cohorts of patients born before 1965 (n = 10,533) and after 1990 (n = 12,502) (Figure 3A). More details can be seen in Supplementary Table S3.

Panels A and B. The boxes indicate the median and quartiles of antibody levels per age (grouped by 2-year strata) and the whiskers indicate the range (excluding outliers). The orange lines visualise the trend (LOESS regression).

In individuals born before 1965 (n = 10,531), median levels remained consistently > 3,000 IU/L and seronegativity was < 5%. In contrast, in individuals born after 1990, median antibody levels peaked at the age of 2 years (962 IU/L; n = 436) and then declined until the age of 10 years (459 IU/L; n = 278). However, no further decline was observed for those aged 11–33 years (n = 9,149), with median antibody levels varying between 375 and 545 IU/L.

**Figure 4:**
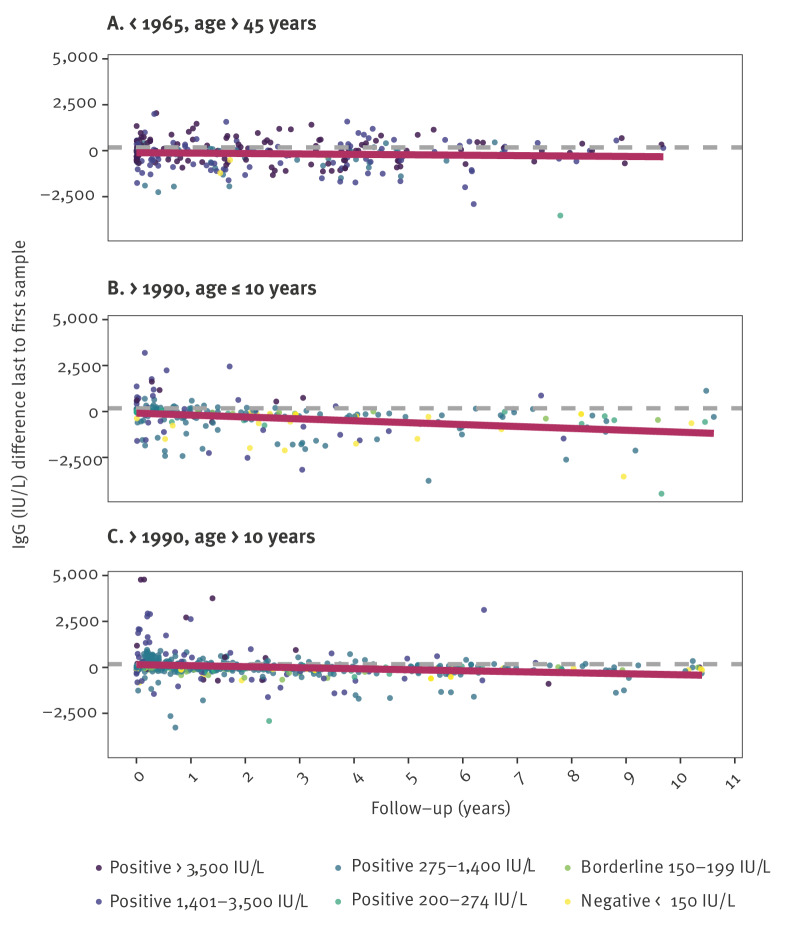
Waning of IgG antibodies against measles virus in follow-up samples from individuals born < 1965 (n = 296) and > 1990 (n = 872), Austria, 2010–2024

Next, we longitudinally analysed waning of antibody levels using the subset of follow-up samples obtained from 1,168 individuals (one follow-up serum per individual) born either before 1965 (n = 296) or after 1990 (n = 872), based on a linear regression analysis of the differences in antibody levels of samples from each individual. There was no statistically significant mean decrease in antibody levels of individuals born before 1965 (estimated decline of around 23 IU/L/year and p = 0.22; Figure 4). More details can be seen in Supplementary Figure S7. Similar to the cross-sectional analysis, in individuals born after 1990, we observed a steeper decline of 98 IU/L/year (p < 0.001) in children aged ≤ 10 years (n = 635), as opposed to a decline of 59 IU/L/year (p < 0.001) for aged > 10 years (n = 237) at the time of the first sample.

Each dot represents the difference between paired antibody samples at the follow-up time between the second and the first samples. The y-axis represents the difference in antibody levels between the second to the first available paired serum samples, while the x-axis shows the follow-up time between the first and second samples (the last available sample for each individual), i.e. each individual is represented by only one dot. The colour-coding of the dots indicates the antibody concentration of the respective last sample. The red lines are linear regression slopes. The panels represent a stratification according to the individual's birth year and age (in years) at the time of the first sample.

### Combined seroprevalence against measles and rubella as a proxy for the vaccination status

To better estimate the proportion of seronegative individuals who were unvaccinated in our study cohort, we analysed the combined serostatus of IgG antibodies against MeV and RuV. Paired MeV and RuV-IgG antibody ELISA results were available from 2,715 individuals born ≥ 1994, the year MMR vaccinations were first implemented in Austria.

In this subset of individuals, we observed a significant correlation between quantitative anti-MeV- and anti-RuV-IgG antibody levels (p < 0.001; Pearson r = 0.48; R^2^ = 0.23), as presented in Supplementary Figure S8. Thus, the serostatus against MeV and RuV was frequently linked. Indeed, among individuals seropositive for MeV, 80.9% were also seropositive for RuV (with 13.5% borderline and 5.6% seronegative), while among seronegative individuals for MeV, 31.9% were seropositive for RuV, 19.5% were borderline, but 48% were seronegative for RuV. The results are presented in Supplementary Table S6.

Finally, we analysed the proportion of seronegative individuals against both viruses as a proxy for the rate of unvaccinated individuals per birth year. Combined seronegativity against both viruses was 6.0% (n = 50) in samples from individuals born 1994–1997 and 6.6% (n = 24) in those born 1998–2001. In samples from individuals born 2002–2017, combined seronegativity ranged between 7.7% (n = 12; 2018–2021) and 11.2% (n = 35; 2010–2013). The results are presented in Supplementary Table S7 and Supplementary Figure S9.

## Discussion

The widespread measles outbreaks in 2023 and 2024 in Austria raised concerns about a considerable decline in measles immunity, possibly due to reduced immunisation rates due to the COVID-19 pandemic, waning antibody levels in vaccinated individuals or existing immunity gaps in certain age groups. We performed this retrospective study to estimate the proportion of anti-MeV-IgG antibody seronegative individuals in a large cohort of > 50,000 individuals.

We identified immunity gaps in large parts of the population, mainly affecting young and middle-aged individuals. Such lack of immunity in specific age groups reflected the age distribution among recent measles cases during outbreaks in Austria, where 545 (84.5%) of 645 measles cases confirmed at the NRL were born ≥ 1990, while 100 cases (14.5%) were born < 1990 (1 January 2023–17 December 2024). Of note, these immunity gaps were even larger in individuals born > 2000 compared with those born 1990–1999. This might be explained by current vaccine hesitancy in Austria, particularly among younger adults, adolescents and parents, as recently shown [15,16].

Consistent with other studies [17,18], we observed a waning in MeV-specific antibody levels to a certain degree over time. Notably, our data indicate that such antibody waning after vaccination was more pronounced during the first years of age, which is expected, as these reflect sampling soon after vaccination. Interestingly, among measles cases confirmed at the NRL of Austria over the past 2 years, where pre-existing immunity to MeV was assessed by measuring antibody avidity, the median age of individuals with high avidity antibodies (indicating reinfection after previous vaccination) was 25.4 years, compared with 18.2 years for those with low avidity or negative IgG at diagnosis. This suggests higher antibody levels in the first years after vaccination provide greater protection.

We did not observe a noticeable effect of the SARS-CoV-2 pandemic on immunisation rates, as there was no specific decrease in the measles seroprevalence rate in children aged < 2 years when birth cohorts 2010–2024 were compared. However, it should be noted that only 102 samples were included.

As the optimal age to vaccinate infants against measles is influenced by maternal antibodies, which may blunt the response to the live virus vaccine [11,19,20], we specifically analysed antibody levels in pregnant people and infants aged < 9 months. Notably, at 3 months, median anti-MeV-IgG antibody levels were < 150 IU/L, i.e. seronegative, corresponding to the rapid waning of maternal anti-MeV antibodies recently demonstrated by others [11]. Furthermore, median antibody levels in pregnant people follow the same trend as in the general population, with lower median antibody titres in pregnant people born after the 1980s compared with those born in earlier decades. Our data thus indicate that, on average, children born after the 1980s will have received gradually lower levels of maternal immunity than those born in earlier decades and are, therefore, likely more vulnerable to measles. It should be noted, however, that few samples were available from pregnant people born before 1973 (n = 49) and from those born after 1998 (n = 33). Conversely, the impact of maternal antibodies on measles vaccine efficacy [11] might be less critical in recently born children than those born in earlier decades. Thus, our data support the recommendation of an earlier initial vaccination dose (e.g. at 6–9 months) in outbreak situations, as is currently recommended in Austria [14].

While the large sample size was a strength of this study, some limitations should be noted. First, a sampling bias cannot be excluded as the analysis was based on samples analysed for diagnostic purposes. However, our results are comparable with similar seroprevalence studies from the region of Tyrol (a federal state of Austria) and the neighbouring countries of Germany and Hungary [21-24]. As the second limitation, the vaccination and infection status of the individuals were unavailable for most of the samples. As seroconversion rates after vaccination are not 100% (especially after a single dose), our seroprevalence estimates are moderately lower than estimates of vaccination rates modelled on vaccine sales [25,26]. Moreover, some individuals born after 1990 could have gained immunity via exposure to MeV, however, as outbreaks are closely monitored by strict contact tracing, we assumed this as a very low proportion of all cases and had a negligible effect on the analysis.

It should be noted that we analysed anti-MeV-IgG antibody levels rather than vaccination coverage. However, to estimate whether the measles seroprevalence in our study cohort might reflect the vaccination status, we examined the proportion of individuals seronegative for MeV who were also seronegative for RuV. Individuals seronegative for MeV were more frequently seronegative for RuV, and proportions of seronegativity against both viruses similarly increased 2002–2017. Altogether, these observations indicate that the measles seroprevalence we assessed in this study might serve as a proxy for the vaccination status.

Nonetheless, it is essential to note that the relationship between MeV (or RuV) antibody levels measured by ELISA and the degree of protection does not directly correlate. Therefore, multiple studies applied neutralisation tests to define a more accurate correlate of protection [13,27]. As a result, low but detectable antibody levels below the cutoff of the ELISA may not necessarily correlate with vulnerability to measles (or rubella) infection.

As current population-level immunity relies on those born in times of widespread measles circulation, the consequences of these immunity gaps will become even more evident in future decades when a more considerable proportion of the population will have been born in the vaccine era. Thus, eliminating or even eradicating measles will become increasingly complex (if not impossible) unless the current vaccination gaps are closed promptly.

## Conclusion

To prevent further measles outbreaks in Austria, the immunity gaps in young and middle-aged individuals identified by this study must be closed quickly, e.g. by catch-up vaccinations of persons without a verified and complete MMR vaccination series. Furthermore, the efforts to vaccinate infants according to the schedule must be increased to counteract further decrease of immunity at the population level due to the continuous decline of the proportion of individuals born during the era of widespread measles circulation.

## Supplementary Data

1543000 Supplementary Material
