# Erratum for Euro Surveill. 2025;30(16)

**DOI:** 10.2807/1560-7917.ES.2024.30.17.250501e

**Published:** 2025-05-01

**Authors:** Eurosurveillance editorial team

**Affiliations:** 1 European Centre for Disease Prevention and Control (ECDC), Stockholm, Sweden

In the article entitled ‘Seroprevalence against measles, Austria, stratified by birth years 1922 to 2024’ by Springer et al., published on 24 April 2025, two birth year intervals were incorrect in Figure 1A and B at the time of publication. This mistake was corrected on 28 April 2025.

