# Supplementary material for: Seroprevalence against measles, Austria, stratified by birth years 1922 to 2024
Source: Euro Surveill. 2025 Apr 24;30(16):2400684. doi: 10.2807/1560-7917.ES.2025.30.16.2400684 (PMC12023729; doi:10.2807/1560-7917.ES.2025.30.16.2400684)
Supplement: Supplementary Material [file 24-00684_WESESLINDTNER_Supplement.pdf]

Supplement to

Seroprevalence against measles, Austria, stratified by birth years 1922-2024

Springer DN, Borsodi C, Camp CV, Redlberger-Fritz M, Holzmann H, Kundi M, Aberle JH, Stiasny K, Weseslindtner L

This supplementary material is hosted by Eurosurveillance as supporting information alongside the article **Seroprevalence against measles, Austria, stratified by birth years 1922-2024** on behalf of the authors, who remain responsible for the accuracy and appropriateness of the content. The same standards for ethics, copyright, attributions and permissions as for the article apply. Supplements are not edited by Eurosurveillance and the journal is not responsible for the maintenance of any links or email addresses provided therein.

Table S1: History of measles, mumps and rubella vaccination recommendations in Austria

Year(s)	Measles vaccinations	Rubella vaccinations	Dose(s)
1963	inactivated MCV vaccine became available and were used in individuals born 1966-1976. [1]		
1967-1969	live attenuated MCV became available [1]		
1974	Nationwide MCV recommended and free of charge for children [1]<br><br>live attenuated bivalent mumps, measles [1, 2]		1st dose at 15 months [2]
1984		Nationwide recommendation for rubella vaccination only for females at 11-13 years of age, monovalent vaccine [3]	
1994	Trivalent MCV (MMR: Measles, Mumps, Rubella) recommended and free of charge for all children [2, 4]	Rubella vaccination recommended for all children, regardless of sex (MMR) [3]	1st dose at age 14-18 months<br>2nd dose at age 6 years [2, 3]
2003			1st dose at age 12 months<br>2nd dose at least 4 weeks later [2]
2012	MMR offered free of charge also for all non-immune adults until the age of 45 years ("catch-up program") [5]		1st dose at age 11 months<br>2nd dose at least 4 weeks later [5]
2017			1st dose at age 10 months<br>2nd dose at least 4 weeks later [6]
Since 2018			Age at first vaccination < 12 months:<br>1st dose at age 10 months<br>2nd dose no less than 3 months later<br><br>Age at first vaccination > 12 months:<br>2nd dose at least 4 weeks later [7, 8]

MCV: measles virus containing vaccine.

Table adapted based on Mutz, Spork 2007 [1], Schmid et al. 2009 [2] and on the respective yearly updates in the national vaccination guidelines ("Österreichischer Impfplan") of the Austrian Ministry of Health [5-8].

Table S2: Possible ELISA cut-offs and corresponding sensitivity and specificity in relation to a positive MeV-NT

Cut-off (IU/l)	Sensitivity (%)	Specificity (%)	Manufacturer interpretation	Manufacturer interpretation (alternative)
≥ 150 IU/l	98.3 %	65.5 %	negative	borderline
≥ 200 IU/l	92.9 %	84.5 %	borderline	positive
≥ 275 IU/l	75.7 %	96.7 %	positive	/

Sensitivity and specificity based on the detection of a positive MeV-NT titer (≥ 10), corresponding to ≥ 120 IU/L calibrated on the WHO NIBSC 97/648 standard.

Table S3: Demographics and anti-measles IgG by year of birth strata

Demographics					Anti-measles IgG				
Year of birth	Individuals (n)	male (%)	female (%)	div./ unk. (%)	median conc. (IU/l)	Q25 conc. (IU/l)	Q75 conc. (IU/l)	% neg. < 200 IU/l	% neg. < 150 IU/l
1922-1925	40	32.5	67.5	0	3267	1615	4269	10	10
1926-1929	68	42.6	57.4	0	3523	2310	4357	2.9	2.9
1930-1933	118	49.2	50.8	0	3488	2151	4349	3.4	3.4
1934-1937	194	42.3	56.7	1	3483	1905	4451	3.1	3.1
1938-1941	499	47.1	52.9	0	3416	1600	4417	2.8	2.6
1942-1945	525	41	59	0	3472	2076	4401	2.3	1.9
1946-1949	778	45.4	54.2	0.4	3506	1835	4475	2.4	2.2
1950-1953	1067	43.1	56.6	0.3	3621	2230	4510	2.4	2.2
1954-1957	1618	40.2	59.6	0.2	3627	2218	4592	2.4	1.8
1958-1961	2486	35.4	64.4	0.2	3584	2133	4467	2.3	1.9
1962-1965	3499	29.6	70.2	0.2	3633	2152	4564	2.4	1.9
1966-1969	4109	27.8	72.1	0.2	3378	1493	4433	3.8	3.0
1970-1973	4181	27.7	72	0.3	2920	857	4270	6.5	5.0
1974-1977	4381	27.1	72.6	0.3	1923	568	3928	10	7.7
1978-1981	4725	26.4	73.3	0.3	879	412	3147	12.6	8.6
1982-1985	4845	25.9	73.7	0.4	627	261	1735	18.3	12.9
1986-1989	4112	28.2	71.5	0.3	530	236	939	19.5	12.9
1990-1993	3616	30	69.7	0.4	544	249	889	17.8	11.0
1994-1997	2899	29.9	69.5	0.7	479	222	835	22.4	14.8
1998-2001	1897	36	63.3	0.8	432	189	777	27	19.0
2002-2005	1448	42.9	56.4	0.7	395	166	766	30	22.4
2006-2009	1188	50	49.2	0.8	574	217	954	23.3	17.6
2010-2013	893	52.2	47	0.8	671	229	1787	22.7	18.3
2014-2017	592	50.8	48.5	0.7	684	272	1711	19.6	15.8
2018-2021	239	57.3	41.4	1.3	738	307	1606	15.1	11.6

Sex: male, female, diverse (div.) or unknown (unk.); conc: concentration; Q25/Q75: 25th and 75th quartliles; % neg.: percent seronegative using <150 IU/l or <200 IU/l as cut-off. Only individuals older than 2 years at the time of sampling were included.

Table S4: Anti-measles IgG by age in individuals born before 1965 or later than 1990

Demographics			Anti-measles IgG				
Year of birth	Age at blood sampling (years)	Individuals (n)	median conc. (IU/l)	Q25 conc. (IU/l)	Q75 conc. (IU/l)	% neg. < 200 IU/l	% neg. < 150 IU/l
<1965	49	61	3548	1840	4415	3.3	3.3
	50	213	3485	1784	4646	4.2	4.2
	51	227	3798	1686	4571	3.5	2.6
	52	285	3689	2053	4597	2.8	2.5
	53	355	3748	2405	4617	2.3	2.3
	54	609	3732	2308	4554	2.1	1.8
	55	679	3721	2241	4655	1.3	0.9
	56	707	3553	2414	4472	1.3	0.7
	57	752	3554	1991	4563	1.9	1.3
	58	672	3676	2118	4511	2.8	2.2
	59	560	3591	2166	4534	1.6	1.4
	60	532	3745	2538	4532	0.8	0.8
	61	465	3526	2091	4423	2.6	1.9
	62	425	3504	2008	4419	1.4	0.9
	63	379	3639	2052	4594	2.9	2.1
	64	362	3612	2119	4453	2.5	2.2
	65	281	3641	2241	4634	1.8	1.8
	66	296	3541	2064	4576	2.4	1.0
	67	256	3438	1978	4487	2.3	2.3
	68	248	3696	1924	4461	1.6	1.2
	69	240	3734	2323	4571	0.8	0.4
	70	220	3582	1811	4450	2.7	1.8
	71	189	3499	1592	4393	2.6	2.6
	72	172	3299	1607	4334	3.5	2.9
	73	151	3381	2209	4516	2	1.3
	74	148	3511	1895	4518	1.4	1.4
	75	139	3568	2008	4422	2.9	2.2
	76	137	3489	2206	4607	1.5	1.5
	77	104	2988	1400	4242	1	1.0
	78	128	3447	2129	4248	1.6	1.6
	79	99	3362	1873	4440	3	3.0
	80	73	3621	1946	4506	0	0
	81	57	3518	2117	4410	0	0
	82	64	3453	1797	4289	3.1	3.1
	83	46	3461	2155	4266	2.2	2.2
	84	46	3434	2011	4331	0	0
	85	36	3423	2138	4021	0	0
	86	31	3776	2542	4395	3.2	3.2
	87	30	3141	1292	4220	10	10.0
	88	16	2806	1521	3859	0	0
	89	14	3422	2132	4814	0	0
	90	9	3364	2986	4224	0	0
	91	9	4149	664	4476	0	0
	92	4	2931	2387	3493	0	0
	93	5	4839	4240	5000	0	3.3
>1990	0	129	186	75	569	51.9	45.7
	1	377	73	50	932	65.3	63.1
	2	436	962	450	2524	17.7	16.7
	3	335	893	395	2856	17.6	14.9
	4	310	829	356	2192	17.1	14.8
	5	323	664	253	1367	20.7	17.3
	6	283	728	379	1443	16.3	13.8
	7	315	588	235	923	22.2	16.8
	8	279	576	215	891	23.7	18.6
	9	288	511	222	961	22.9	18.1

	10	278	459	178	833	28.1	23.4
	11	249	505	219	852	22.1	17.7
	12	266	382	163	814	31.2	22.2
	13	276	401	193	755	26.1	16.3
	14	341	439	175	816	27.6	21.4
	15	358	437	192	836	28.2	19.3
	16	400	443	210	889	24.5	17.8
	17	436	488	189	839	27.3	18.6
	18	549	432	189	793	26.6	18.4
	19	594	482	225	789	21.2	15.7
	20	696	455	206	796	24	16.2
	21	669	485	222	845	21.5	13.2
	22	651	491	225	803	22.6	15.2
	23	675	493	231	864	20.4	12.9
	24	582	484	239	815	18.9	11.9
	25	503	416	218	758	23.1	14.5
	26	472	457	204	774	24.2	13.3
	27	408	511	215	852	23.8	15.2
	28	352	452	213	832	23.6	16.8
	29	265	463	209	765	24.2	17.4
	30	181	492	200	816	25.4	15.5
	31	126	456	224	754	21.4	18.3
	32	85	545	243	847	20	10.6
	33	15	375	134	737	40	33.3

Age at sampling: rounded to full years. conc: concentration; Q25/Q75: 25th and 75th quartliles; % neg.: percent seronegative using <150 IU/l or <200 IU/l as cut-off. Two individuals older than 93 were not reported to maintain anonymization.

Table S5: Demographics and anti-measles IgG by year of birth strata of pregnant people

Year of birth	Individuals (n)	median conc. (IU/l)	Q25 conc. (IU/l)	Q75 conc. (IU/l)	% neg. < 200 IU/l	% neg. < 150 IU/l
1966-1969	12	3455	1772	4384	0	0
1970-1973	37	3394	922	4560	8.1	5.4
1974-1977	111	993	373	3282	14.4	9.0
1978-1981	252	722	269	2756	20.2	11.5
1982-1985	392	571	250	1364	20.7	12.8
1986-1989	407	454	234	833	24.8	9.3
1990-1993	309	454	226	796	21.7	11.3
1994-1997	139	385	193	770	27.3	15.1
1998-2001	33	284	142	531	36.3	33.3

conc: concentration; Q25/Q75: 25th and 75th quartliles; % neg.: percent seronegative using <150 IU/l or <200 IU/l as cut-off.

Table S6: Qualitative Measles IgG and Rubella IgG ELISA results of individuals born in/since 1994

Measles IgG (Interpretation)	Rubella IgG (Interpretation)	Individuals (n)	Percent (by MeV-IgG result)
positive	positive	1687	80.9 %
	borderline	282	13.5 %
	negative	117	5.6 %
borderline	positive	102	59.3 %
	borderline	42	24.4 %
	negative	28	16.3 %
negative	positive	146	31.9 %
	borderline	89	19.5 %
	negative	222	48.6 %

Table S7: Qualitative Measles IgG and Rubella IgG ELISA results of individuals born since 1994 by year of birth

Year of birth	Number of individuals by combination of MeV-IgG and RV-IgG results (n)				Percentage of individuals by year of birth and results (%, row-wise)			
	One or both positive	Both borderline	One negative, one borderline	Both negative	One or both positive	Both borderline	One negative, one borderline	Both negative
1994-1997	725	15	41	50	87.2 %	1.8 %	4.9 %	6.0%
1998-2001	318	9	14	24	87.1 %	2.5 %	3.8 %	6.6 %
2002-2005	306	5	21	35	83.4 %	1.4 %	5.7 %	9.5 %
2006-2009	327	8	18	34	84.5 %	2.1 %	4.7 %	8.8 %
2010-2013	260	3	14	35	83.3 %	1 %	4.5 %	11.2 %
2014-2017	259	1	6	32	86.9 %	0.3 %	2%	10.7 %
2018-2021	139	1	3	12	89.7 %	0.7 %	1.9 %	7.7 %

MeV: measles virus, RV: rubella virus.

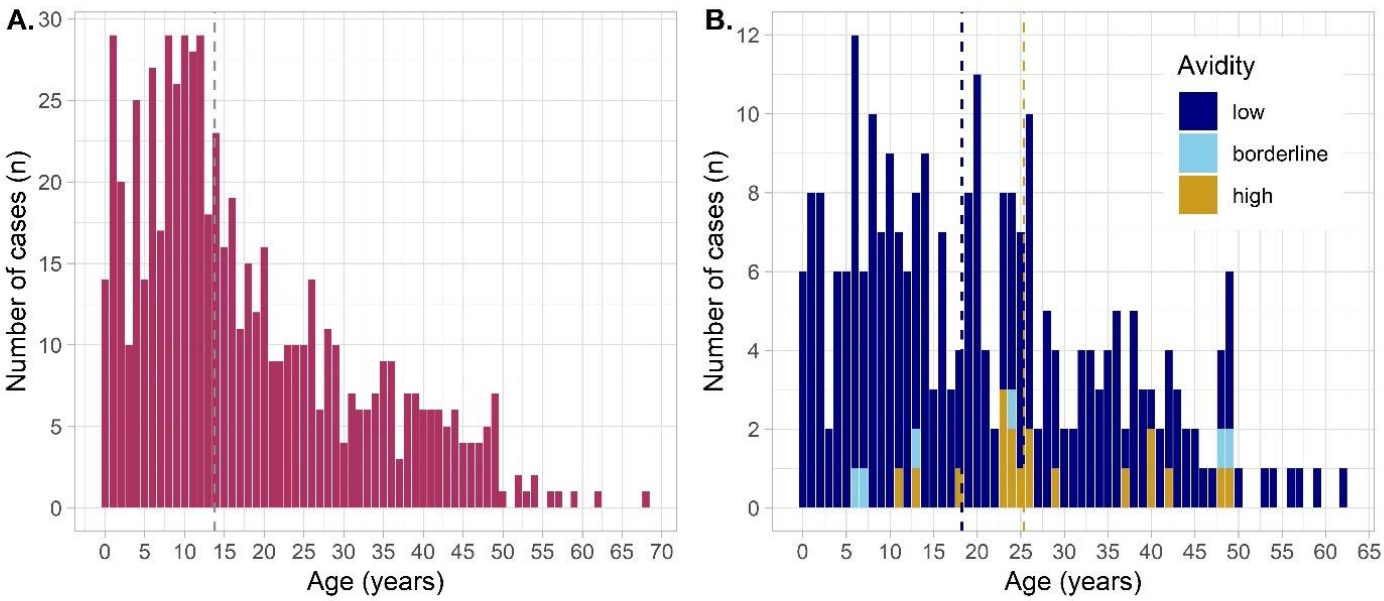

Figure S1: Age distribution of measles cases confirmed at the National Reference Lab in Austria in 2023 and 2024

Panel A. shows a histogram of the age distribution (in years) of A. the measles cases confirmed at the national reference lab (n = 645) and B. of the confirmed cases, for which serum samples were available (n = 268), stratified by IgG antibody avidity (as indicated by the color-coding). Sera with negative/borderline IgG (pre-seroconversion) were included in those with negative avidity. The dashed lines indicate the median age at sample collection in A. for all cases (n = 645): 13.8 years; B. low avidity (n = 241): 18.2 years, borderline avidity (n = 6): 18.2 years, high avidity (n = 18): 25.4 years). n = 3 cases with invalid avidity results were excluded.

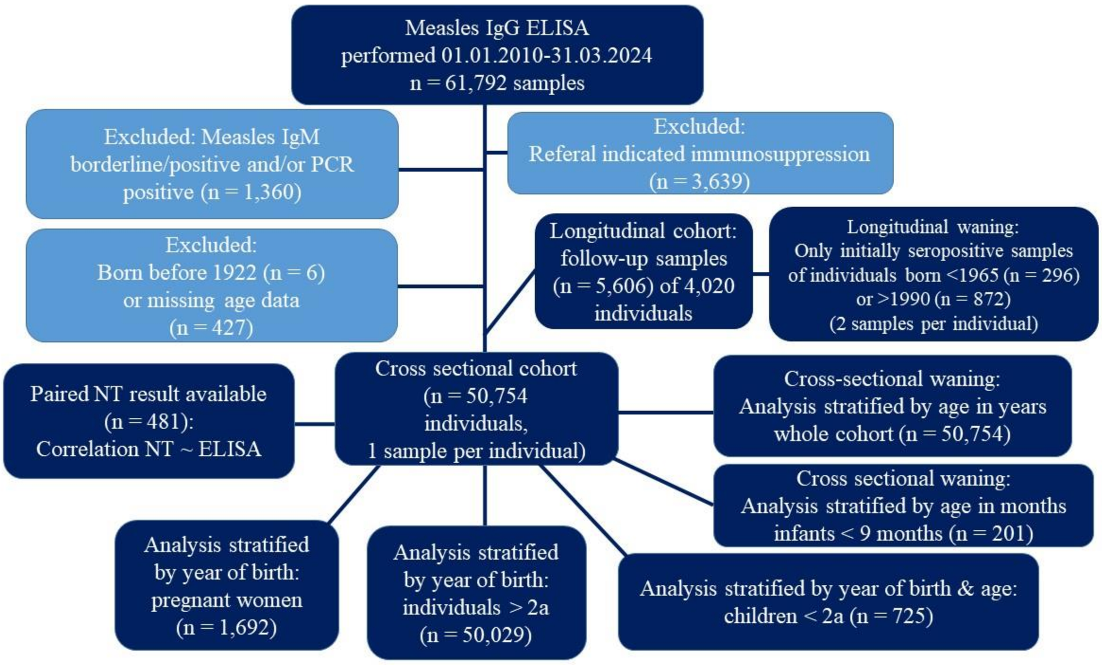

Figure S2: Inclusion and analysis study plan.

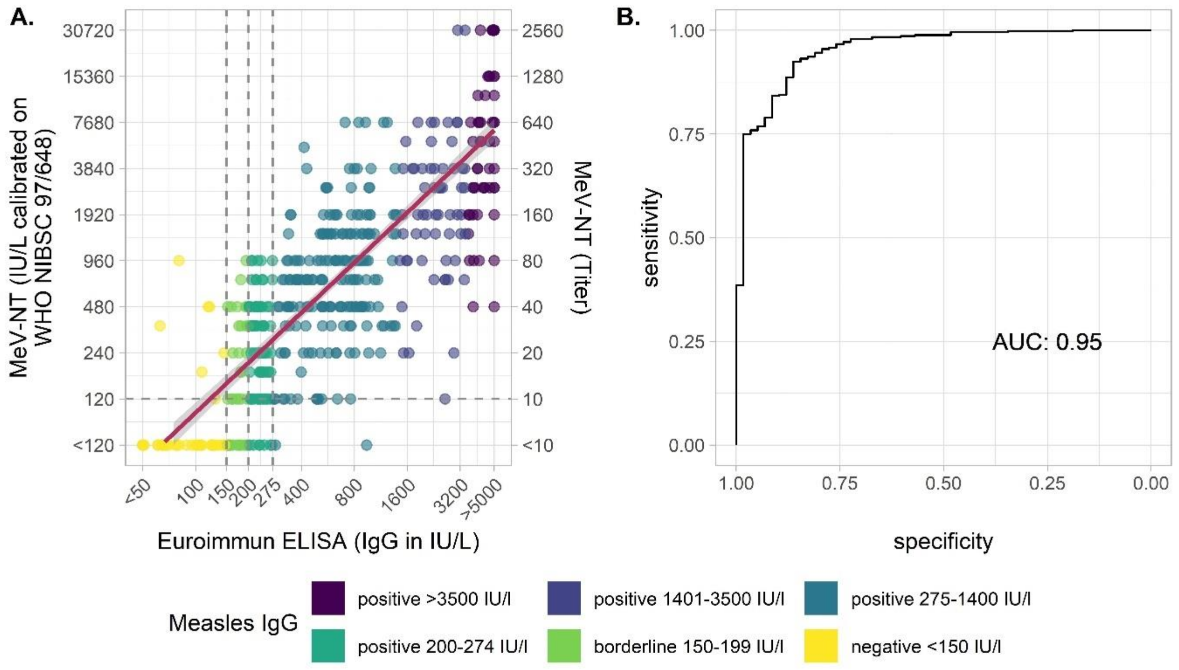

Figure S3: Correlation of ELISA results to MeV-NT titer

A. Antibody concentrations (IU/l) as assessed by the ELISA and corresponding neutralization test results in IU/L 120 IU/L calibrated on the WHO NIBSC 97/648 standard or in titer (right y-axis) of a subset of samples where both results were available (n = 481). Each dot represents a single serum, colored according to the legend given below. The dashed lines represent possible cut-off values. B. Receiver operating characteristic (ROC) analysis of ELISA values (in IU/l) corresponding to a positive NT titer (≥ 10 or 120 IU/L). The area under the curve (AUC) is noted in the panel. MeV-NT: Measles Virus neutralization test

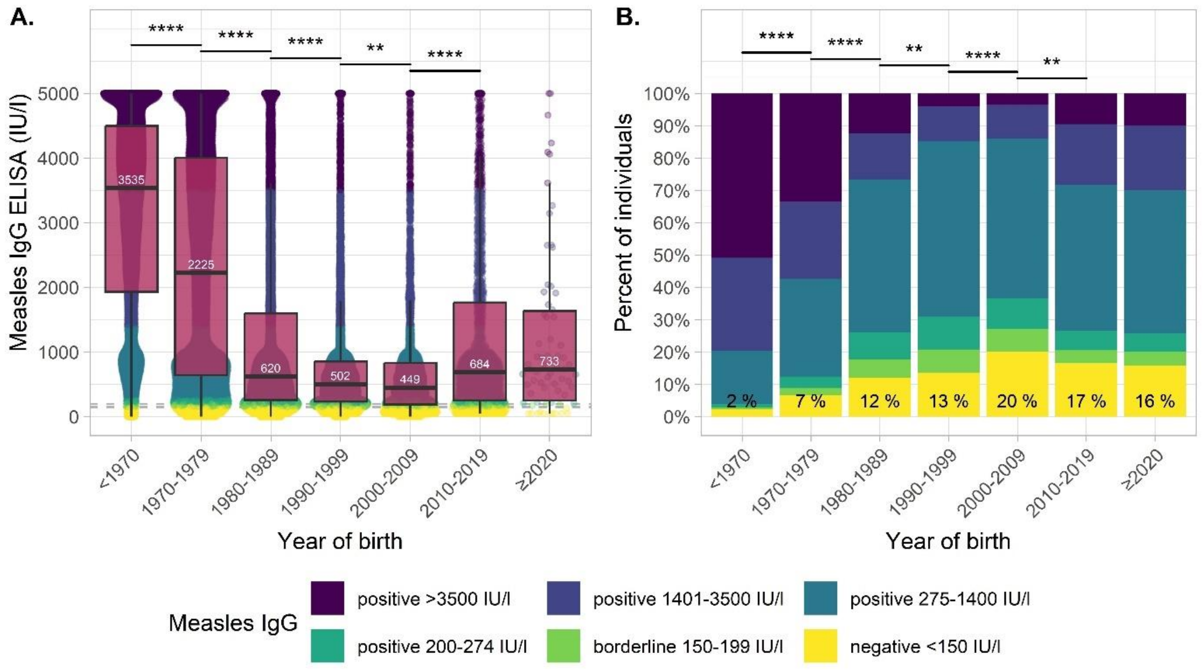

Figure S4: Anti-measles seroprevalence by decade of birth (data from Figure 1)

The figure displays results from 50,029 measles IgG tests of individuals aged 2-93 years sampled between 2010 and 2024. A. Anti-measles IgG in IU/l of individuals stratified according to a year of birth prior to 1970 and then in decades until 2019, or in/later than 2020. The boxes indicate the median and the quartiles, and the whiskers indicate the range (excluding outliers). Mean values are additionally indicated in white font for each group. The dots represent single sera, and their placement approximates the data distribution. B. Percentages of different strata of positive and negative samples per year-of-birth stratum as indicated by the colors provided in the legend. The percentage values within the bars indicate the percentages of negative samples by group (<150 IU/l). A. & B.: Significance values for all significant comparisons are represented by stars: ** $p < 0.01$, **** $p < 0.0001$ in a Kruskal-Wallis test followed by multiplicity adjusted Dunn's test (A.) or Pairwise Chi-Square tests followed by Bonferroni correction (B.).

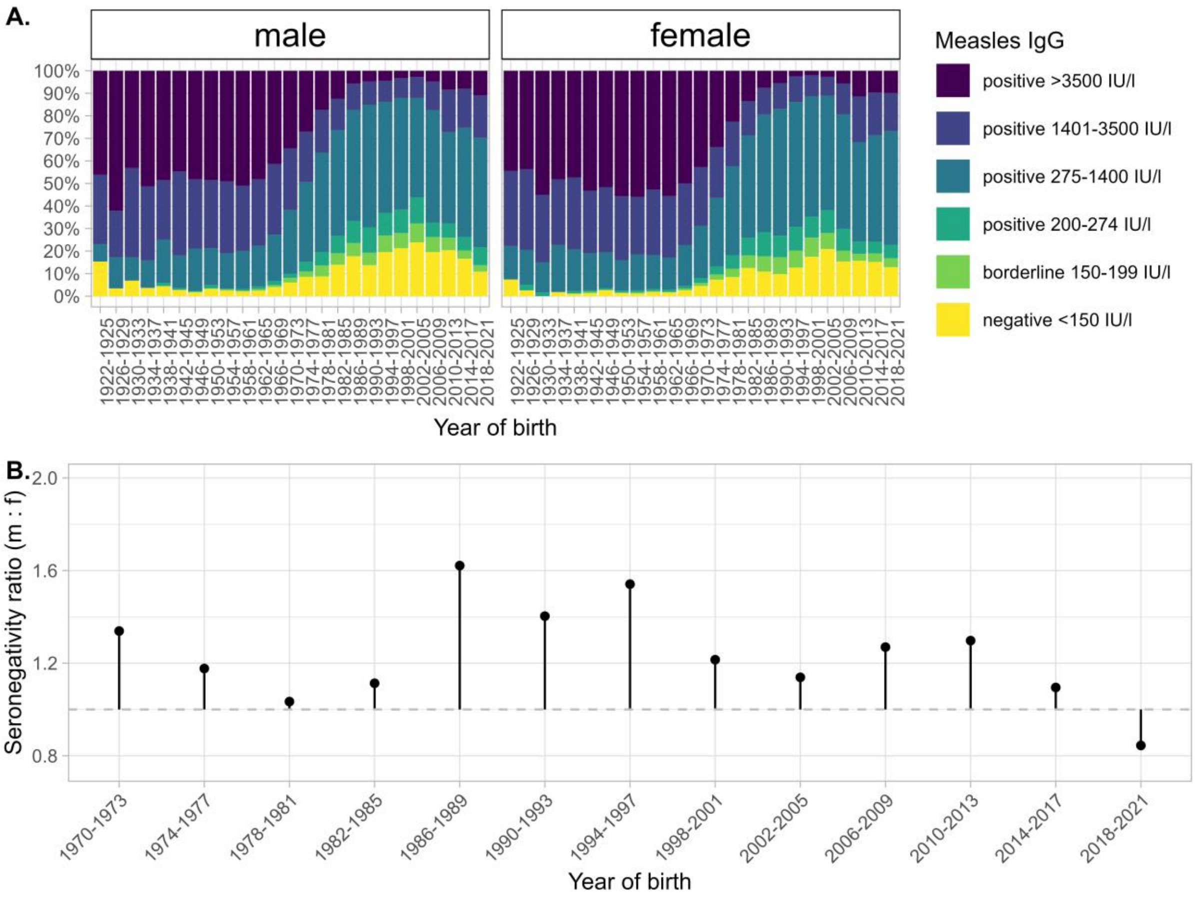

Figure S5: Anti-measles seroprevalence in male vs. female individuals (data from Figure 1)
A. Seroprevalence in % stratified by 4-year year of birth strata. The colored bars indicate the percentages of individuals per the year-of-birth that are seropositive and negative for measles IgG, color-coded for different antibody concentrations (as given in the legend). B. Ratio of the percentage of seronegative individuals per year-of-birth stratum in males compared to the percentage in females. The dashed line indicates a ratio of 1. Values above the dashed line indicate that a larger proportion of males were seronegative compared to females, and vice-versa. m: male; f: female (sex)

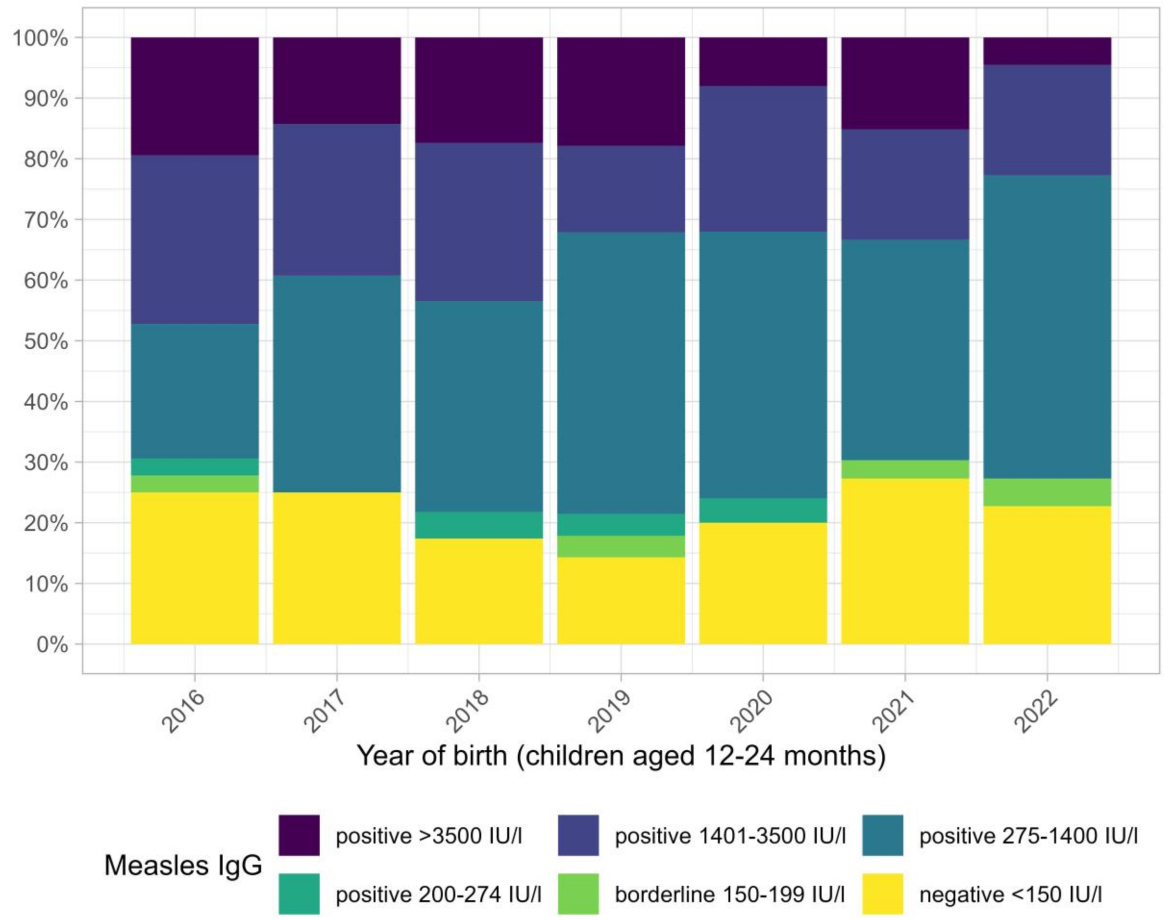

Figure S6: Anti-measles seroprevalence in children aged 12-24 months

Seroprevalence in % stratified by the year of birth of children aged between 12-24 months at the date of sample collection (n = 195). The colored bars indicate the percentages of individuals that are seropositive and negative for measles IgG, color-coded for different antibody concentrations (as given in the legend). The recommended date of vaccination in Austria for the MMR-vaccine is at 9 months of age (2 doses) [8], thus, children born later than july 2019 should have been scheduled for their first vaccination later than march, 2020 (i.e. during the SARS-CoV-2 pandemic).

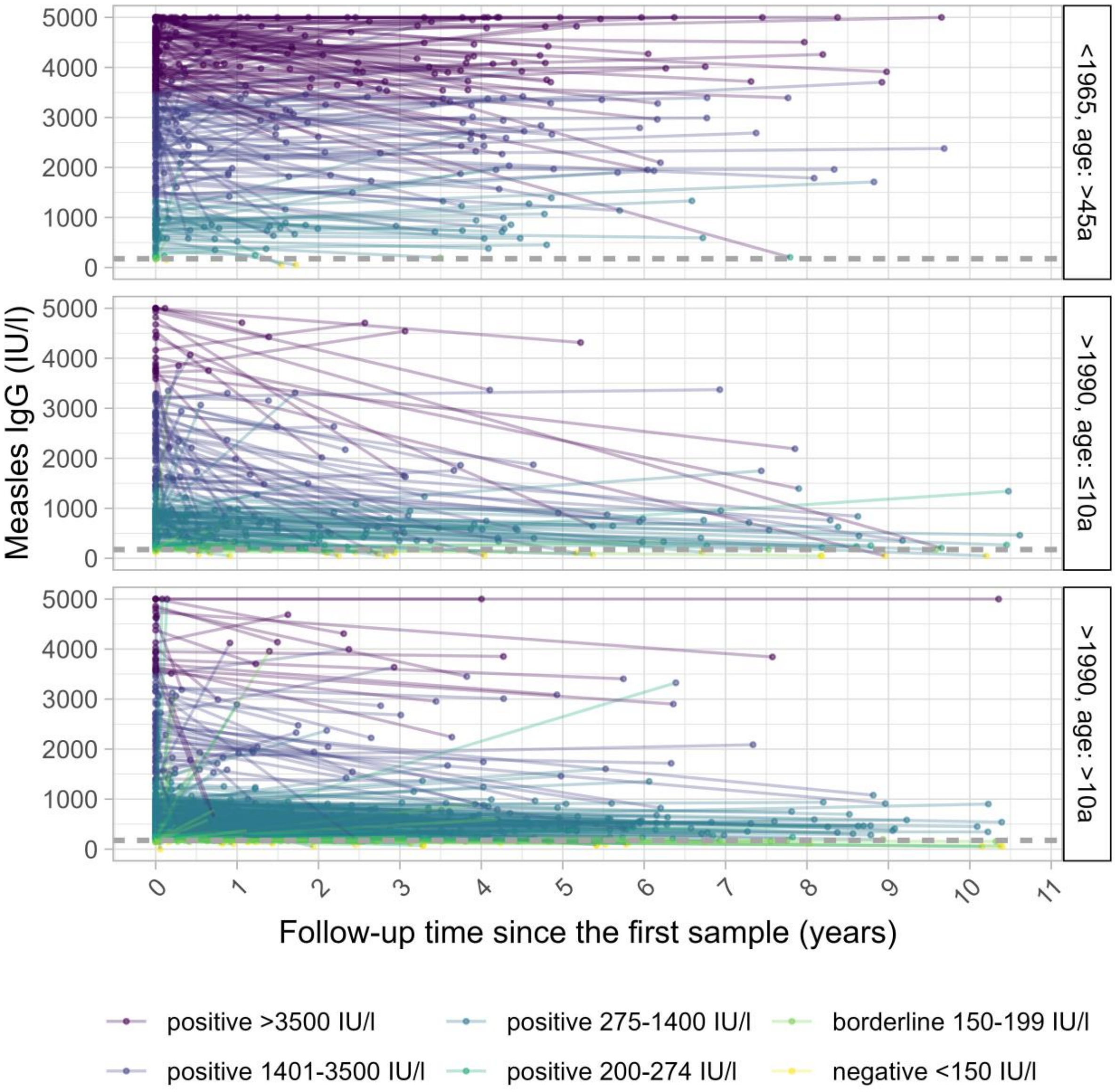

Figure S7: Anti-measles antibody kinetics kinetics (data from Figure 4)

Antibody levels for each individual (two samples per individual, of n = 1168 individuals) are represented by dots connected by lines. The date of the first sample is defined as timepoint 0; the x-axis shows the follow-up time since the first sample. The color-coding indicates the different antibody concentrations of the respective sample as given in the legend. The panels represent a stratification according to the individual's year of birth (before 1965, immunity presumably due to measles infection or after 1990, immunity presumably due to vaccination), as well as the age (in years (a)) at the time of the first sample. The dashed lines indicate the thresholds for "negative" (<150 IU/l) and "borderline (<200 IU/l), respectively.

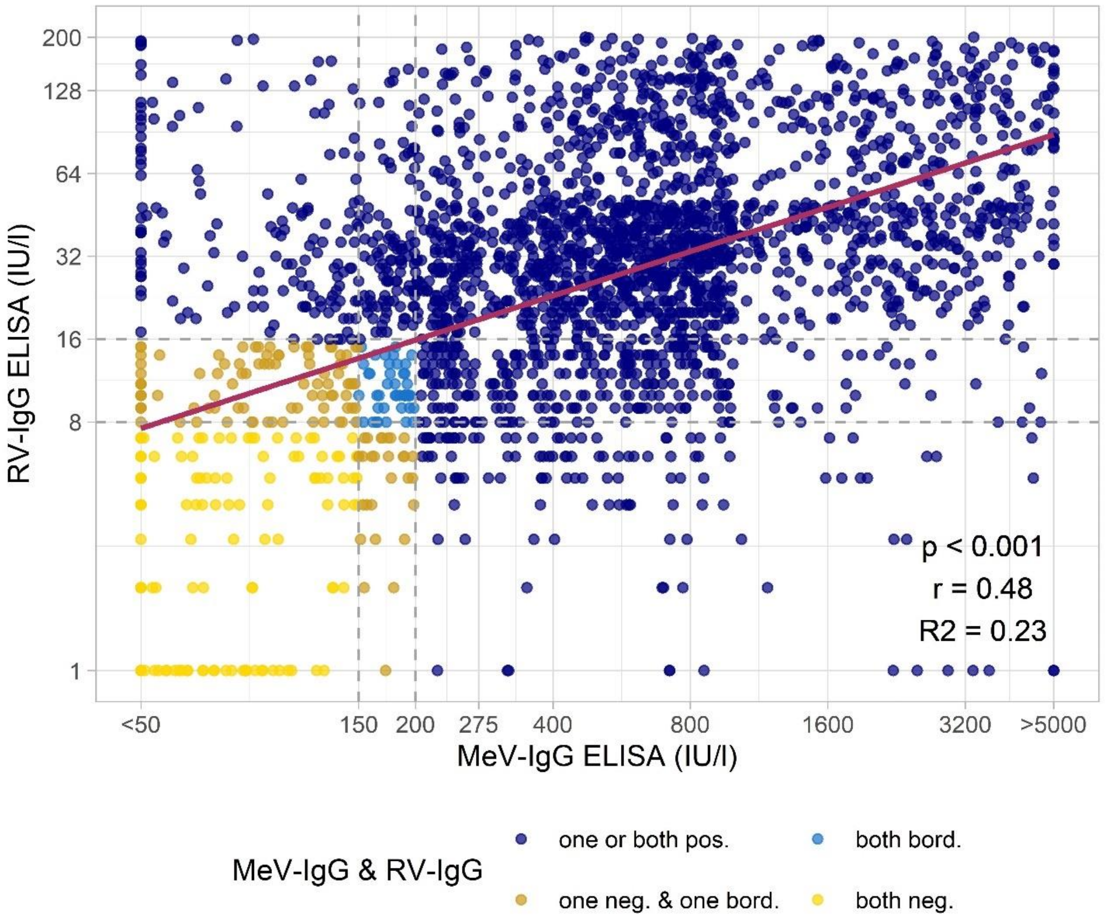

Figure S8: Correlation between Anti-measles virus IgG and Anti-rubella virus IgG ELISA results The figure shows data from n = 2715 individuals born in/after 1994, for which paired measles virus (MeV) and rubella virus (RV) IgG results were available (i.e., a subset of the individuals shown in Figure 1). Each point represents results from a single individual. The dashed lines indicate the respective thresholds for "negative" and "borderline" (MeV-IgG: <150 IU/l and <200 IU/l; RV-IgG: <8 IU/l and <16 IU/l). The color summarizes a combined interpretation. The red line indicates the slope of a linear regression (after log2-transformation) and the corresponding p-value, Pearson's r and R^2 are noted in the figure. MeV: Measles virus. RV: Rubella virus.

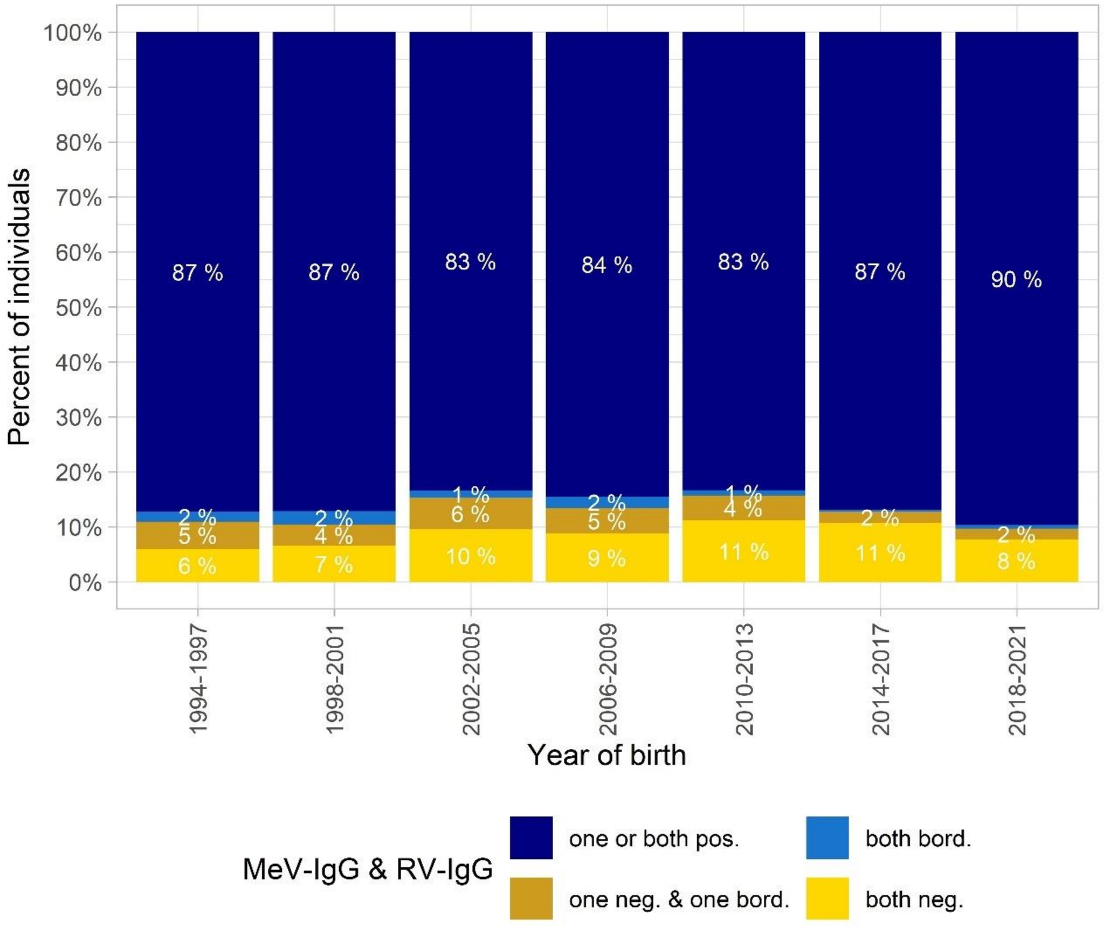

Figure S9: Proportion of combined Anti-Measles and Anti-Rubella IgG ELISA seronegativity
The figure shows data from n = 2715 individuals born in/after 1994, for which paired Measles virus IgG and Rubella virus IgG results were available (i.e., a subset of the individuals shown in Figure 1). The colored bars show the proportions of combined Anti-Measles- and Anti-Rubella-IgG results stratified by the year of birth. Dark blue indicates that at least one of the two ELISA results was positive regardless of the second result (one or both pos.), light blue indicates that both results were borderline (both bord.), orange indicates that one result was negative and one result was borderline (one neg. / one bord.), yellow indicates that both MeV-IgG and RV-IgG results were negative. The respective percentages within each year-of-birth stratum are noted in white font. Pos.: positive. Bord.: borderline. Neg.: negative. MeV: Measles Virus. RV: Rubella Virus.

Supplementary methods

Live-virus neutralization test

Neutralization tests were performed as previously described [9]. Serial two-fold dilutions of serum samples, starting from 1:10, were incubated with 50-100 TCID50 measles virus strain B3 for one hour at 37 °C. This virus-serum mixture was then applied to a monolayer of Vero-SLAM cells and incubated for four days before microscopically assessing a cytopathic effect (CPE). The final titer was determined by the inverse of the last dilution at which no CPE was observed. Titers ≥ 10 were considered positive, and dilution series were performed up to 1:2560. Using the 3rd WHO NIBSC 97/648 standard for measles, 3000 IU/L corresponded to a geometric mean titer of 250 in five separate repetitions. Thus, a positive MeV-NT titer (≥ 10) corresponds to ≥ 120 IU/L.

Quantification of rubella specific antibodies

Serum IgG antibodies directed against Rubella virus (RV) were quantified using a commercial enzyme-linked immunosorbent assay (ELISA) following the manufacturer's instructions (Anti-Rubella Virus ELISA IgG, Euroimmun, Lübeck Germany). For the qualitative interpretation, the cutoffs recommended by the manufacturer were used (<8: negative, ≥8: borderline, ≥ 16: positive)

Rubella vaccination & circulation in Austria

From 1984 to 1993, rubella vaccination was only recommended for girls aged 11-13 with the aim of preventing congential rubella syndrome and thus the circulation of rubella virus was not prevented. In 1994, nationwide rubella vaccination for all children (regardless of sex) was introduced (2-dose MMR scheme), aiming to eliminate measles circulation (see Table SX). For the years between 1994 and 2007, only limited data are available for the incidence of rubella in Austria as no systematic surveillance was in place. In 2007, rubella became a notifiable disease in Austria, and in 2007 to 2024, rubella has not been circulating widely in Austria with the exception of sporadic outbreaks (e.g. in 2008 [3]).

Analysis of combined measles and rubella seronegativity

As both measles and rubella circulation was low in the last two decades (or at least since 2007), the presence of measles- and/or rubella-specific antibodies of individuals born in that period can be interpreted as an indication of a past vaccination with the MMR vaccine. Inversely, the proportion of individuals negative for antibodies against both viruses could be assumed to better reflect the actual proportion of unvaccinated individuals as opposed to those who have lost their immunity. Thus, we first selected the subset of our study population born after 1994 for which paired measles virus IgG (MeV-IgG) and rubella virus IgG (RV-IgG) ELISA results were available (n = 2715). First, a linear regression was used to assess antibody level correlations. Then, percentages of dual-virus serostatus groups (positive, borderline and negative) were calculated and stratified by birth year. Finally, we calculated the percentages of individuals who were positive against at least one of the virus, who were borderline against both viruses, who were borderline and negative, or who were negative against both viruses.

Statistical software: R Version 4.2.0; R-Studio 2022.02.3; R-Packages: cowplot, dplyr, scales, stringr, lubridate, pROC, ggbeeswarm, ggplot2, scales, viridis

References used in the Supplement

1. Mutz I, Spork D. [History of recommendations for immunizations in Austria]. Wien Med Wochenschr. 2007;157(5-6):94-7.
2. Schmid D, Holzmann H, Alfery C, Wallenko H, Popow-Kraupp TH, Allerberger F. Mumps outbreak in young adults following a festival in Austria, 2006. Euro Surveill. 2008;13(7).
3. Schmid D, Kasper S, Kuo HW, Aberle S, Holzmann H, Daghofer E, et al. Ongoing rubella outbreak in Austria, 2008-2009. Euro Surveill. 2009;14(16).
4. Schmid D, Holzmann H, Schwarz K, Kasper S, Kuo HW, Aberle SW, et al. Measles outbreak linked to a minority group in Austria, 2008. Epidemiol Infect. 2010;138(3):415-25.
5. Bundesministerium für Soziales G, Pflege und Konsumentenschutz, (Hg.) B. Impfplan Österreich 2012. 2012.
6. Bundesministerium für Soziales G, Pflege und Konsumentenschutz, (Hg.) B. Impfplan Österreich 2017. 2017.
7. Bundesministerium für Soziales G, Pflege und Konsumentenschutz, (Hg.) B. Impfplan Österreich 2018. 2018.
8. Bundesministerium für Soziales G, Pflege und Konsumentenschutz (BMSGPK). Impfplan Österreich 2023/2024 Version 2.0. 2024.
9. Semmler G, Aberle SW, Griebler H, Richter L, Schmid D, Stiasny K, et al. Performance of Four IgM Antibody Assays in the Diagnosis of Measles Virus Primary Infection and Cases with a Serological Profile Indicating Reinfection. Journal of clinical microbiology. 2021;59(5).
